# EP29 A rare case of marginal zone lymphoma of the breast complicating primary Sjögren’s syndrome

**DOI:** 10.1093/rap/rkaa052.028

**Published:** 2020-11-03

**Authors:** Alaeldin Mohamednour, Mohsin Hussein, Afifa Riaz, James Taylor

**Affiliations:** 1 Leicester Royal Infirmary, Leicester, United Kingdom; 2 Northampton General Hospital, Northampton, United Kingdom

## Abstract

**Case report - Introduction:**

Primary Sjögren’s patients are a higher risk of developing non-Hodgkin lymphoma (NHL) compared with patients with other autoimmune disorders and to the general population.

Parotid and submandibular salivary glands are the most frequent localization of MALT lymphomas in pSS. Here we report a case of marginal zone lymphoma of the breast in a patient with long-standing primary Sjögren’s, and we will discuss the clinical and serological predictors for the development of lymphoma.

**Case report - Case description:**

A 79-year-old female with a 19-year history of primary Sjögren’s syndrome (anti-Ro, anti-La positive) was reviewed in the rheumatology clinic for progressive worsening of sicca symptoms. Her past medical history included recurrent pulmonary emboli, osteoporosis of the spine, asthma, microscopic colitis, Ischemic heart disease and hypothyroidism. She was a non – smoker and consumed minimal alcohol. Her past surgical history included excision of her left salivary gland, 10 years ago. She was undergoing investigation for a new left breast mass for which a biopsy revealed possible involvement by marginal zone lymphoma. A PCR was performed which was equivocal. Subsequently, she developed a right breast lump and an ultrasound scan showed a well-defined 23 x 10 mm oval hyperechoic lesion in the right upper quadrant and a 29 x 9 mm right axillary lymph node. A staging CT chest abdomen and pelvis and bone marrow sample revealed localized disease. Biopsy of breast lump was performed with PCR analysis. This revealed morphological and immunophenotypical appearances most consistent with a low-grade B –Cell lymphoma and favour a marginal zone lymphoma.

The patient was reviewed by an oncologist and was treated with 24 Gy radiotherapy in 12 daily fractions and followed up by the advanced practitioner.

**Case report - Discussion:**

Primary Sjögren’s Syndrome is an autoimmune disease characterised by lymphocytic infiltration of exocrine glands which can manifest in specific organs or as a systemic illness. Patients are at elevated risk of developing lymphoproliferative diseases including non-Hodgkin's lymphoma with a reported prevalence of 5%. On histological analysis, most patients demonstrate low-grade marginal zone B cell lymphoma with approximately 85% occurring in extranodal locations including the parotid and submandibular glands. Some patients may go on to develop high-grade lymphoma.

Concerning overall disease activity, it has been recently demonstrated that a stable moderate/high disease activity, calculated either with the EULAR Sjögren’s syndrome disease activity index (ESSDAI) or with the ClinESSDAI, an ESSDAI variant excluding the biological domain, was independently associated with subsequent lymphoma occurrence.

Symptomatic cryoglobulinaemic vasculitis (CV) is observed in about 3–4% of pSS patients and has been linked to the development of lymphoma. Other clinical markers of lymphoma such as palpable purpura, low levels of C4, lymphocytopenia, low levels of IgM, elevated levels of β2-microglobulin

Malignant proliferation has been reported in the literature and, to the best of our knowledge, only two case reports of a marginal zone lymphoma of the breast complicating primary Sjögren’s syndrome exist.

**Case report - Key learning points:**

1/ This case emphasises the need for careful clinical examination in this exceedingly rare entity.

2/ Although parotid and submandibular gland are commonest extranodal site for NHL, other organs such as thyroid, ovaries and breast might be rarely be affected.

3/ Clinicians should be alert if there are some clinical and serological features such as cryoglobulin and persistently low complement which usually predicts the development of lymphoma.

